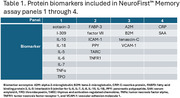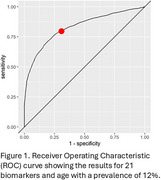# Development and cross‐validation of a proteomic blood test to rule out Alzheimer's Dementia in primary care

**DOI:** 10.1002/alz70861_108593

**Published:** 2025-12-23

**Authors:** Steve Hunsucker, Melissa Petersen, Rajesh Nandy, David Julovich, Daniel Tessier, Heather Giles, Sid E. O'Bryant

**Affiliations:** ^1^ Cx Precision Medicine, Fort Worth, TX USA; ^2^ Institute for Translational Research, University of North Texas Health Science Center, Fort Worth, TX USA; ^3^ University of North Texas Health Science Center, Fort Worth, TX USA

## Abstract

**Background:**

The increasing elderly population and consequent increase in people with memory loss and dementia is creating immense resource demands on diagnostic pathways for Alzheimer’s Dementia (AD). The causes of memory loss are many and a test that is easy to use and interpret could be of great value in primary care to discriminate between those unlikely to have AD and those requiring referral to specialist AD diagnostic centers. O’Bryant et al have extensively described the use of blood tests using multiplexed proteomic assays and machine learning algorithms to identify or rule out neurodegenerative diseases. This report describes the development and cross‐validation of a rule‐out test for AD (NeuroFirst™Memory).

**Method:**

A four panel, 21 biomarker (Table 1) proteomic assay was developed for use on the Luminex multiplex immunoassay platform. The serum assay was analytically characterized by determining, for each analyte, lower limits of detection and quantification, linearity, precision and accuracy. Clinical cross‐validation used biorepository serum samples from 80 subjects with clinically diagnosed AD and 475 cognitively normal controls, aged over 55 years. Serum analyte concentrations (pg/ml) for each sample were measured. The biomarker data and age were used to retrain and test a previously developed Random Forest algorithm, using a 70:30 training/test split to provide estimates of accuracy, sensitivity, specificity, negative and positive predictive values (NPV, PPV). An AD prevalence of 12% was selected, based on that expected in the target primary care population.

**Result:**

Analytical characterization parameters for linearity, precision and accuracy met pre‐specified criteria and the test kits and assay were deemed to be acceptable to proceed to clinical cross‐validation. Across all clinical samples (*N* =555) and all 21 biomarkers, 99.8% of biomarker concentrations fell within the detectable range of the assay, making it appropriate to perform the Random Forest analysis. The algorithm yielded a Receiver‐Operating Characteristic curve (Figure 1) with an AUC of 0.831. Accuracy was 0.740, sensitivity 0.693, specificity 0.777, PPV 0.315 and NPV 0.949.

**Conclusion:**

The results demonstrate that the NeuroFirst™ Memory test could provide a simple blood test to rule out AD. Next steps are formal analytical and clinical validation in preparation for use in primary care.